# A Multi-Stage Framework for Kawasaki Disease Prediction Using Clustering-Based Undersampling and Synthetic Data Augmentation: Cross-Institutional Validation with Dual-Center Clinical Data in Taiwan

**DOI:** 10.3390/bioengineering12070742

**Published:** 2025-07-07

**Authors:** Heng-Chih Huang, Chuan-Sheng Hung, Chun-Hung Richard Lin, Yi-Zhen Shie, Cheng-Han Yu, Ting-Hsin Huang

**Affiliations:** 1 Department of Computer Science and Engineering, National Sun Yat-sen University, Kaohsiung 80424, Taiwan; d093040003@student.nsysu.edu.tw (H.-C.H.);; 2 Division of Cardiology, Department of Internal Medicine, Kaohsiung Chang Gung Memorial Hospital, Kaohsiung 83301, Taiwan

**Keywords:** Kawasaki disease, class imbalance, clustering, ensemble learning, data augmentation

## Abstract

Kawasaki disease (KD) is a rare yet potentially life-threatening pediatric vasculitis that, if left undiagnosed or untreated, can result in serious cardiovascular complications. Its heterogeneous clinical presentation poses diagnostic challenges, often failing to meet classical criteria and increasing the risk of oversight. Leveraging routine laboratory tests with AI offers a promising strategy for enhancing early detection. However, due to the extremely low prevalence of KD, conventional models often struggle with severe class imbalance, limiting their ability to achieve both high sensitivity and specificity in practice. To address this issue, we propose a multi-stage AI-based predictive framework that incorporates clustering-based undersampling, data augmentation, and stacking ensemble learning. The model was trained and internally tested on clinical blood and urine test data from Chang Gung Memorial Hospital (CGMH, n = 74,641; 2010–2019), and externally validated using an independent dataset from Kaohsiung Medical University Hospital (KMUH, n = 1582; 2012–2020), thereby supporting cross-institutional generalizability. At a fixed recall rate of 95%, the model achieved a specificity of 97.5% and an F1-score of 53.6% on the CGMH test set, and a specificity of 74.7% with an F1-score of 23.4% on the KMUH validation set. These results underscore the model’s ability to maintain high specificity even under sensitivity-focused constraints, while still delivering clinically meaningful predictive performance. This balance of sensitivity and specificity highlights the framework’s practical utility for real-world KD screening.

## 1. Introduction

Artificial intelligence (AI) and machine learning (ML) are increasingly applied in modern healthcare for early disease detection and clinical risk prediction, offering clinicians timely decision support based on complex medical data. These technologies enable earlier identification of underlying health threats and facilitate more precise, targeted interventions, ultimately mitigating disease progression and alleviating the burden on healthcare systems. However, a major obstacle to deploying AI in clinical settings lies in the intrinsic imbalance of medical data. In most datasets, positive (minority class) cases are vastly outnumbered by negative (majority class) cases—a challenge that is especially pronounced in the context of rare diseases and significantly hampers model performance and clinical utility.

Kawasaki disease (KD), also known as mucocutaneous lymph node syndrome [1,2], exemplifies this issue. First described by the Japanese pediatrician Tomisaku Kawasaki in 1961 [1], KD primarily affects children under five years of age, with peak incidence between 18 and 24 months and a slight male predominance [1]. Despite decades of investigation, its etiology remains elusive. Diagnosis is predominantly clinical, based on features such as prolonged fever (5 days or more), erythema and desquamation of the extremities, polymorphous rash, mucosal changes (e.g., “strawberry tongue,” cracked lips), non-purulent conjunctivitis, and cervical lymphadenopathy. However, many patients present with incomplete or atypical symptoms, increasing diagnostic complexity [1,3]. Without timely treatment—most critically, if intravenous immunoglobulin (IVIG) is not administered within 10 days of onset—patients are at risk of coronary artery aneurysms, long-term cardiac sequelae, or even death [1,3]. KD therefore remains a significant challenge in pediatric diagnosis and care, with no available preventive strategies to date [2,3].

Efforts to apply ML in KD prediction are frequently undermined by the disease’s low prevalence. Traditional models often overfit the majority class, resulting in poorly calibrated decision boundaries and diminished ability to identify positive cases. This imbalance elevates the risk of missed or delayed diagnoses, severely limiting the models’ clinical applicability. Thus, enhancing sensitivity and stability in detecting rare cases is vital for building reliable diagnostic support systems.

Recent developments in data augmentation—particularly synthetic data generation—offer promising avenues for addressing these challenges [4,5,6,7,8]. By simulating the statistical properties of real-world cases, synthetic data can increase sample diversity and improve representation of minority classes without distorting underlying distributions. These enhancements can lead to better learning of underrepresented patterns, improved generalization, and greater robustness when applied to varied clinical environments. In this study, we propose a multi-stage AI framework that combines data augmentation and ensemble learning to construct a predictive model for KD using routine laboratory and demographic data. Beyond optimizing model performance within a single institution, we emphasize cross-institutional generalization to accommodate variations in clinical workflows, laboratory standards, and patient populations. Data were collected from two major medical centers in CGMH and KMUH for training, internal testing, and external validation. This study proposes a predictive framework that integrates clustering-based undersampling (CBU) with three advanced synthetic data generation techniques—Conditional Tabular GAN (CTGAN)  [9], Kernel Density Estimation (KDE)  [10], and GAN-based Bayesian Logistic Regression (GANBLR)  [11]—coupled with a stacking ensemble learning strategy, aiming to deliver a robust and generalizable pipeline suitable for clinical deployment. Recent high-impact studies have increasingly underscored the transformative potential of artificial intelligence in the diagnosis of rare diseases, not only facilitating variant prioritization in genetic disorders but also demonstrating wide-ranging applicability across healthcare systems  [12,13,14].

To clarify the scope of this study, we define the following objectives and hypotheses:

**Primary Objective:** To develop an AI framework that enhances sensitivity and cross-institutional generalizability for Kawasaki disease (KD) prediction under conditions of severe class imbalance.

**Secondary Objective:** To evaluate the impact of various undersampling and data augmentation strategies—including clustering-based filtering and synthetic sample generation—on overall model performance.

Our primary hypothesis is that combining CBU with GAN-based data augmentation will improve model stability and specificity under high-recall thresholds. We further hypothesize that each augmentation method (CTGAN, KDE, GANBLR) will exhibit distinct generalization characteristics depending on the heterogeneity of the dataset.

The intended clinical application of this study is a decision-support tool that leverages routine laboratory tests (blood and urine analyses) to assist pediatricians in the early identification of KD, particularly in resource-limited settings or among patients presenting with atypical clinical features.

## 2. Materials and Methods

### 2.1. Data Sources

Clinical data were obtained from two leading medical centers in Taiwan: Chang Gung Memorial Hospital (CGMH) spanning from 2010 to 2019 and Kaohsiung Medical University Hospital (KMUH) spanning from 2012 to 2020. The study cohort comprised pediatric patients aged 0 to 5 years who were clinically diagnosed with KD [1,2]. The control group consisted of age-matched febrile children who did not meet the diagnostic criteria for KD.

Ethical approval was obtained from the Institutional Review Board (IRB-2025-001), and all patient data were fully anonymized prior to analysis. The dataset included 22 features categorized into three groups: demographic characteristics (sex, age, and month of visit), 17 routine blood test parameters (e.g., white blood cell count, red blood cell count, hemoglobin, platelet count, C-reactive protein), and 2 urinalysis indicators (urinary white blood cells and pyuria). An overview of feature categories is provided in Table 1.

### 2.2. Preprocessing

The original dataset exhibited pronounced class imbalance, with KD cases substantially underrepresented at an approximate ratio of 1:64. Training classification models directly on such imbalanced data would likely skew predictions toward the majority class, impairing the model’s ability to identify positive cases. To mitigate this issue, we developed a two-stage data processing pipeline that integrates CBU with augmentation data generation. The impact of various strategy combinations was systematically evaluated to optimize model performance.

In the undersampling stage, we compared the performance of conventional random undersampling (RUS) with that of CBU. Both approaches were paired with CTGAN-based augmentation to ensure a consistent experimental setup. The results demonstrated that CBU, by removing clusters with extreme imbalance or consisting solely of negative cases, more effectively preserved representative samples while filtering out low-informative boundary noise, thereby enhancing downstream classification stability [15,16,17].

To optimize clustering performance, three commonly used distance metrics were evaluated within the CBU framework: Euclidean distance, representing straight-line proximity in multidimensional space; Manhattan distance, defined by the sum of absolute differences across dimensions; and Cosine distance, which captures angular similarity between vectors irrespective of magnitude. These metrics represent distinct similarity paradigms and were assessed for their impact on clustering robustness. Among them, Euclidean distance consistently produced the most stable results across both internal and external validation datasets and was thus selected as the default metric for all subsequent procedures [18,19].

With Euclidean-CBU established as the fixed undersampling approach, we next evaluated the performance of three data augmentation techniques—CTGAN, KDE, and GANBLR—in expanding the size and diversity of the minority class [9,10,11,20]. Clinical records were obtained from two major medical centers in Taiwan. CGMH contributed a total of 74,641 samples, which were partitioned into training and test sets in an 8:2 ratio. The training set comprised 913 KD-positive and 58,799 KD-negative samples, while the test set included 229 positives and 14,700 negatives. The KMUH dataset, consisting of 62 positive and 1520 negative samples, was reserved exclusively for independent validation. No feature selection or normalization was applied, as all variables represent standardized laboratory test values. Consequently, no risk of data leakage due to preprocessing within cross-validation folds was present. These distributions are summarized in Table 2.

**Figure 1:**
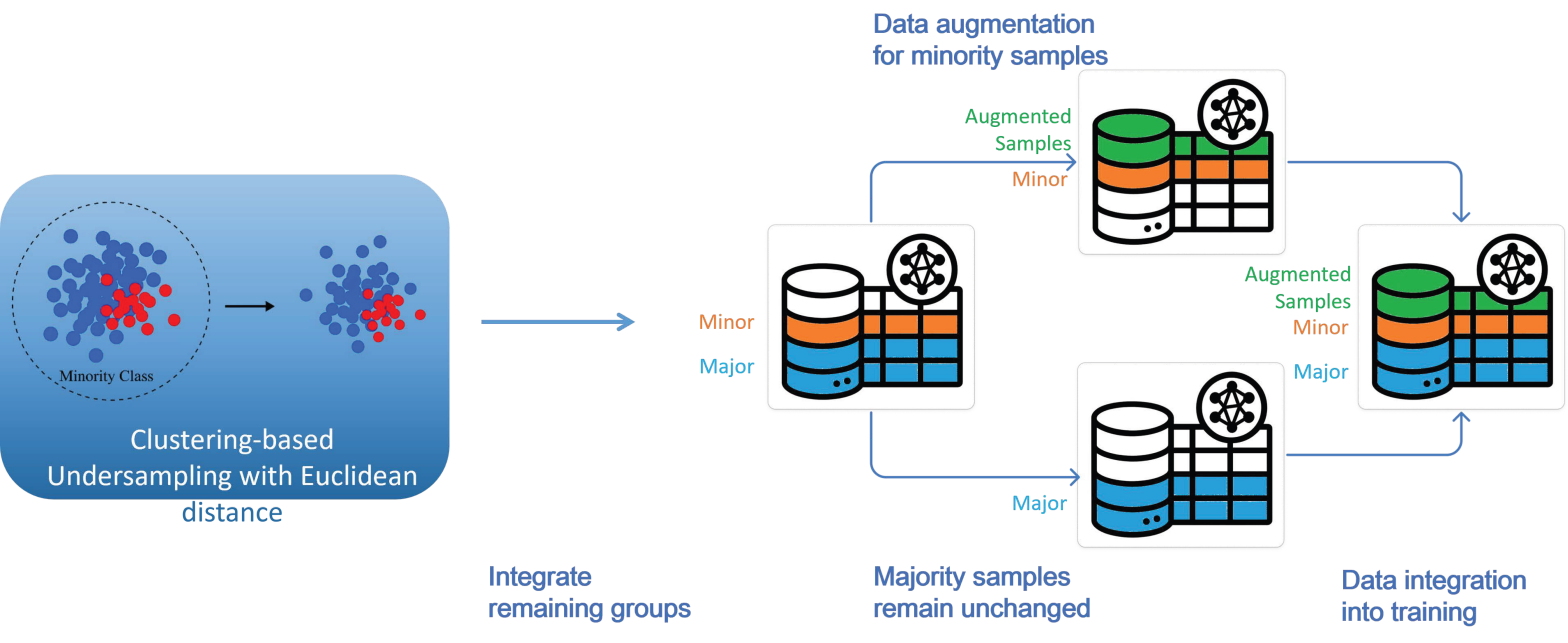
Preprocessing workflow.

The preprocessing workflow is illustrated in Figure 1. K-means++ clustering was performed using each of the three distance metrics [15]. Clusters exhibiting extreme imbalance (e.g., KD-to-non-KD ratio > 1:200) or containing only negative samples were excluded to remove low-informative or redundant data. This initial filtering step improved the class ratio to approximately 1:59. Detailed statistics under each clustering metric are presented in Table 3.

Although undersampling improved class balance, clinical applications demand high sensitivity. To further enhance detection of minority-class instances, synthetic KD samples were generated using the three augmentation techniques and combined with the real data to reach a target ratio of 1:24.

**Table 3:** Post-clustering and augmentation statistics by distance metric.

Distance Metric	Original Positives	Retained Negatives	Augmented Samples	Final Positives	Pos/Neg Ratio
Euclidean	913	52,454	1296	2186	1:24
Manhattan	913	52,287	1290	2178	1:24
Cosine	913	43,817	951	1826	1:24

To assess the statistical quality of the synthetic data, we employed two widely used metrics: Maximum Mean Discrepancy (MMD), which evaluates global distributional similarity, and Pairwise Correlation Difference (PCD), which measures the preservation of inter-variable relationships [21,22]. This evaluation strategy aligns with a recently proposed comprehensive framework for validating synthetic tabular health data, which identifies data fidelity, structural consistency, and downstream utility as foundational pillars for trustworthy deployment [23]. A detailed comparison is provided in Section 3.4.

The number of synthetic samples required to achieve the target class ratio (e.g., 1:24) was calculated using

(1) $$N_{\mathrm{aug}}=\frac{N_{\mathrm{major}}}{R_{\mathrm{target}}}-N_{\mathrm{minor}},\;R_{\mathrm{target}}=24,$$

### 2.3. Model Design

**Figure 2:**
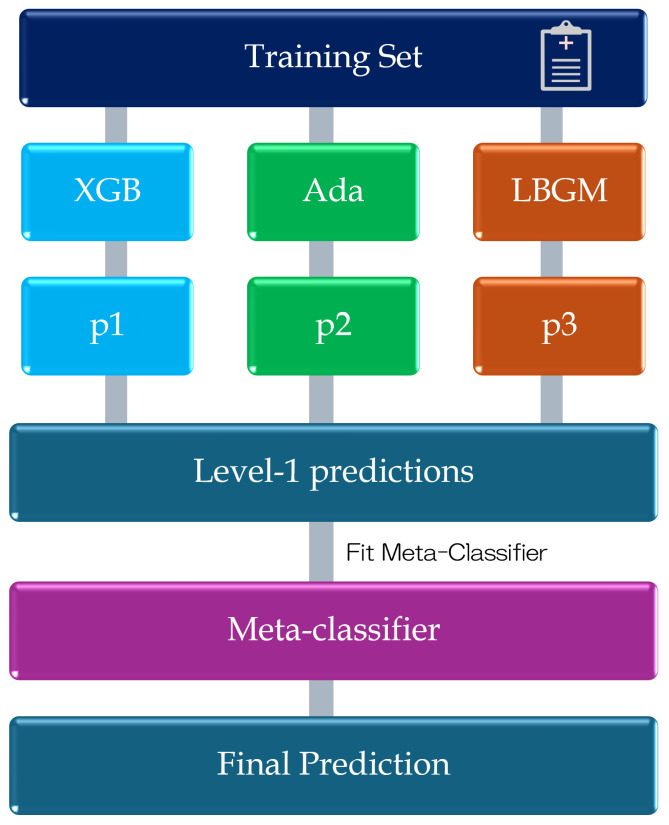
Stacking ensemble model combining XGBoost, AdaBoost, and LightGBM, with logistic regression as the meta-classifier.

Ensemble learning integrates multiple models to boost predictive performance. Among available strategies, stacking employs a meta-classifier that synthesizes the outputs of several base classifiers, forming a robust architecture known as a stacking ensemble model. This approach effectively harnesses the complementary strengths of diverse algorithms. In our framework, XGBoost (learning rate = 0.1, maximum depth = 6) [24], AdaBoost (31 leaf nodes, feature subsampling rate = 0.8) [25], and LightGBM (50 boosting iterations) [26] are adopted as base classifiers, with logistic regression serving as the meta-classifier with its regularization parameter set to C=1.0. To enhance both predictive accuracy and model generalizability, particularly under high-recall constraints, we implemented this stacking ensemble structure, as depicted in Figure 2.

We selected these three base classifiers for their proven performance with structured biomedical data and complementary learning characteristics. XGBoost and LightGBM are efficient gradient boosting frameworks that handle missing values well and can model complex nonlinear relationships. AdaBoost, a classical boosting method, focuses on misclassified instances and remains robust against noisy data. Furthermore, all three models are widely adopted in clinical machine learning workflows due to their favorable balance of predictive power, computational efficiency, and interpretability. While more complex models such as deep neural networks may offer advantages in modeling spatial or sequential data, they typically require larger training samples and offer limited transparency—making them less suitable for this study’s context involving structured, tabular laboratory test data [27]. For these reasons, deep models were not adopted in this work.

The entire model was trained using five-fold cross-validation [28], with hyperparameters optimized at each stage to mitigate overfitting and improve the model’s sensitivity to minority-class instances. This model structure offers a balance between flexibility and reproducibility, making it suitable for deployment in varied clinical scenarios.

To improve clarity and transparency, the full data preprocessing and augmentation pipeline is presented in Algorithm 1, including clustering-based undersampling, synthetic data generation, and stacking ensemble training. Although random seeds were not explicitly specified during dataset partitioning or preprocessing, all procedures were conducted using the default behavior of the corresponding open-source libraries. Given the intrinsic variability of clinical datasets across institutions, the objective of this study is to assess the robustness and generalizability of the proposed framework, rather than to enforce exact replication of any single experimental run.
**Algorithm 1** KD Classification Training Pipeline with Clustering-Based Undersampling and Data Augmentation.**Input:** KD training dataset $D_{\mathrm{train}}$; augmentation method G∈{CTGAN,KDE,GANBLR}**Initialization:** K-means++ clustering (k=10, Euclidean distance); augmentation ratio 1:24; base classifiers B={XGBoost,AdaBoost,LightGBM}**Output:** Trained stacking ensemble model1:**Step 1: Clustering-Based Undersampling**2:Apply K-means++ clustering to $D_{\mathrm{train}}$ with k=103:**for** each cluster $C_{i}$, i=1 to 10 **do**4:    **if** $C_{i}$ is pure negative or imbalance ratio > 1:200 **then**5:        Discard $C_{i}$6:    **end if**7:**end for**8:Let $D_{\mathrm{filtered}}\leftarrow ⋃$ of retained clusters9:**Step 2: Data Augmentation**10:Extract minority samples $D_{\mathrm{minority}}$ from $D_{\mathrm{filtered}}$11:Compute $N_{\mathrm{aug}}=\left\lfloor \frac{|D_{\mathrm{major}}|}{24}\right\rfloor -\left|D_{\mathrm{minority}}\right|$12:Generate $D_{\mathrm{synth}}=G\left(D_{\mathrm{minority}},N_{\mathrm{aug}}\right)$13:Combine $D_{\mathrm{filtered}}$ and $D_{\mathrm{synth}}$ into $D_{\mathrm{balanced}}$14:**Step 3: Stacking Model Training**15:Split $D_{\mathrm{balanced}}$ into 5 folds16:**for** each fold k=1 to 5 **do**17:    $D_{\mathrm{train}}^{\left(k\right)}\leftarrow D_{\mathrm{balanced}}\setminus {\mathrm{fold}}_{k}$18:    $D_{\mathrm{val}}^{\left(k\right)}\leftarrow {\mathrm{fold}}_{k}$19:    **for** each classifier b∈B **do**20:        Train *b* on $D_{\mathrm{train}}^{\left(k\right)}$21:        Predict on $D_{\mathrm{val}}^{\left(k\right)}$, store predicted scores ${\hat{y}}^{\left(k,b\right)}$22:    **end for**23:**end for**24:Aggregate ${\hat{y}}^{\left(k,b\right)}$ into meta-feature matrix $\hat{Y}$25:Train meta-classifier (Logistic Regression) using $\hat{Y}$26:**return** trained stacking model

### 2.4. Evaluation Criteria

Model evaluation is a critical component for validating both clinical utility and algorithmic reliability. We assessed model performance using a confusion matrix-based framework incorporating key classification metrics: recall (sensitivity), specificity, precision (positive predictive value, PPV), negative predictive value (NPV), F1-score, and F2-score. The F1-score represents the harmonic mean of precision and recall and is best suited for scenarios where both are equally prioritized. The F2-score, by contrast, applies a weighted harmonic mean that emphasizes recall (80%) over precision (20%), aligning more closely with clinical applications where high sensitivity is critical, particularly in the early detection of rare conditions [29].

Given the elevated clinical risk and low incidence of KD, this study prioritized maximizing the detection of true positive cases. To this end, we used the F2-score as a supplemental metric to evaluate model stability under high-recall conditions. In addition, we systematically compared the predictive performance of various data processing strategies, including the use of raw data, data augmentation alone, and a hybrid approach combining CBU with incremental synthetic sample generation [20]. We also examined the effects of different combinations of distance metrics and synthesis techniques on model output.

To ensure robustness across varying clinical thresholds, model evaluation was conducted at multiple recall targets (80%, 85%, 90%, and 95%), enabling a comprehensive analysis of the proposed framework’s generalizability and stability.

All model development and evaluations were conducted on a workstation running Ubuntu 20.04.4 LTS, equipped with an NVIDIA GeForce RTX GPU, Santa Clara, CA, USA. The software environment included Python libraries such as scikit-learn, SciPy, NumPy, pandas, XGBoost, and SDV.

## 3. Results

### 3.1. Overall Framework Performance

We evaluated a unified predictive framework that integrates CBU, data augmentation, and a stacking ensemble model. Performance was assessed using internal test data from CGMH and externally validated on an independent dataset from KMUH. The primary goal was to optimize predictive accuracy under high-recall conditions while ensuring robust generalization across institutions.

As summarized in Table 4, the proposed framework achieved the highest F1-score on the CGMH test set (67.6%), outperforming both the baseline XGBoost model trained on raw data (65.8%) and the stacking ensemble model without augmentation (66.3%). It also marginally outperformed the stacking ensemble model trained with data augmentation alone (67.5%). On the external KMUH dataset, due to the extreme rarity of KD cases, the original XGBoost model almost completely failed to detect the minority class (with 0% specificity). However, the proposed framework maintained strong performance, achieving an F1-score of 50.7%, a value substantially higher than the baseline model (7.5%) and the CTGAN-only approach (39.7%), thus highlighting its superior cross-institutional generalizability. This demonstrates its potential utility in real-world screening applications across varied healthcare environments.

**Figure 3:**
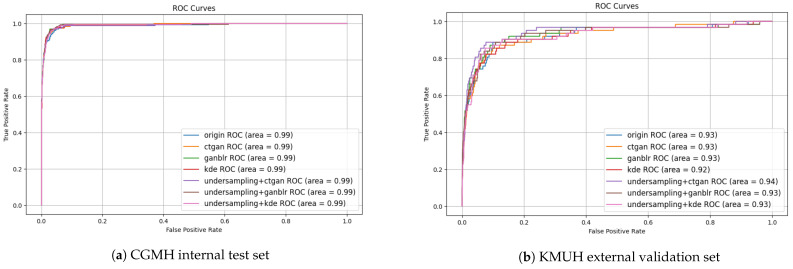
ROC curves comparing augmentation strategies with and without undersampling. GANBLR achieves the highest AUC across both CGMH and KMUH.

**Figure 4:**
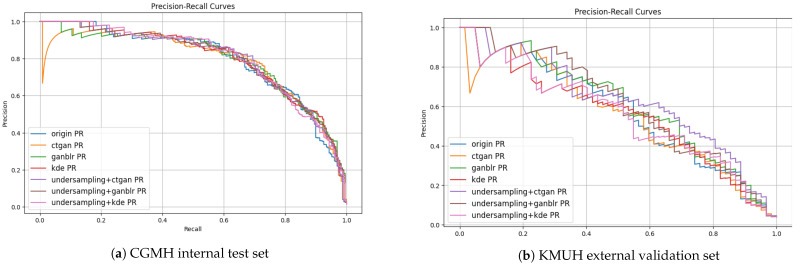
Precision–recall curves illustrating model performance under class imbalance. GANBLR and CTGAN retain higher precision across recall levels.

To further support the quantitative metrics reported in Table 4, we generated Receiver Operating Characteristic (ROC) and precision–recall (PR) curves (Figure 3 and Figure 4), which offer a visual summary of classifier behavior under different combinations of data augmentation and sampling approaches on both the CGMH and KMUH datasets.

The ROC curves demonstrate that all models incorporating synthetic augmentation outperformed the baseline. Notably, the GANBLR-augmented model achieved the highest area under the curve (AUC) values—approximately 0.99 for CGMH and 0.93 for KMUH—indicating strong discriminative power and generalizability across institutions. The PR curves, which are particularly informative under class imbalance, illustrated the trade-off between precision and recall. Although a drop in precision was observed on the external KMUH dataset, both GANBLR and CTGAN models—especially when paired with undersampling—maintained elevated precision across mid-to-high recall intervals.

**Table 4:** Comparison of overall framework performance (recall = 85%).

Method	F1-Score (%)	PPV (%)	Specificity (%)
CBU + GANBLR + Stacking (CGMH)	67.6	56.0	98.9
CBU + CTGAN + Stacking (KMUH)	50.7	35.1	93.8
XGBoost (Original Data, CGMH)	65.8	53.3	98.8
XGBoost (Original Data, KMUH)	7.54	3.92	0
Stacking (Original Data, CGMH)	66.3	54.1	98.9
Stacking (DA only, CTGAN, CGMH)	67.5	55.0	91.1
Stacking (DA only, CTGAN, KMUH)	39.7	24.8	89.2

Together, these graphical results reinforce the tabular findings and present compelling visual evidence for the proposed framework’s robustness and applicability in clinical settings.

To further evaluate the model’s behavior under class-imbalanced conditions, we selected the GANBLR + Stacking configuration and computed the confusion matrices (Table 5) at a fixed recall threshold of 85%. This threshold was chosen because it offers a more stable and interpretable balance between sensitivity and precision. Although higher recall settings (e.g., 90%) can also achieve strong sensitivity, they lead to substantial drops in both precision and specificity—particularly on the external KMUH dataset—due to a sharp increase in false positives. In contrast, the 85% threshold maintains sufficient recall while preserving better precision and specificity across both CGMH and KMUH datasets. As such, we consider this setting to be a more stable and representative benchmark for assessing the model’s generalization under cross-institutional data heterogeneity. The corresponding confusion matrices are presented below and discussed further in Section 4.4.

### 3.2. Impact of Undersampling and Augmentation Strategies

To better understand the contribution of individual components to overall model performance, we conducted a series of experiments to systematically evaluate the effects and stability of different undersampling strategies, clustering distance metrics, and data augmentation techniques. With CTGAN selected as the fixed data augmentation method, we compared RUS and CBU using datasets from both CGMH and KMUH. As shown in Table 6, when recall was fixed at 95%, CBU consistently outperformed RUS in terms of F1-score, precision, and specificity. In particular, CBU improved the F1-score by up to 12.5 percentage points on the external KMUH dataset, highlighting its effectiveness in preserving informative samples while filtering out noise. We further assessed the performance of CBU using three commonly used clustering distance metrics: Euclidean, Manhattan, and Cosine.

Table 7 presents the results under each distance metric. Euclidean distance consistently produced the best or second-best results on both the CGMH and KMUH datasets, indicating its superior stability [18]. Based on this observation, Euclidean distance was adopted as the default setting in our subsequent analyses.

For the data augmentation module, we compared three techniques—CTGAN, KDE, and GANBLR—under the same Euclidean-based CBU configuration. As shown in Table 8, although all three methods performed well on the CGMH test set, CTGAN yielded the best results on the external KMUH dataset, suggesting stronger generalizability to heterogeneous data sources [9,10,11].

Overall, the results of this section suggest that combining CBU with generative augmentation—particularly CTGAN or GANBLR—is key to enhancing both cross-institutional robustness and model performance in this study.

### 3.3. Effect of Augmentation Ratios

**Figure 5:**
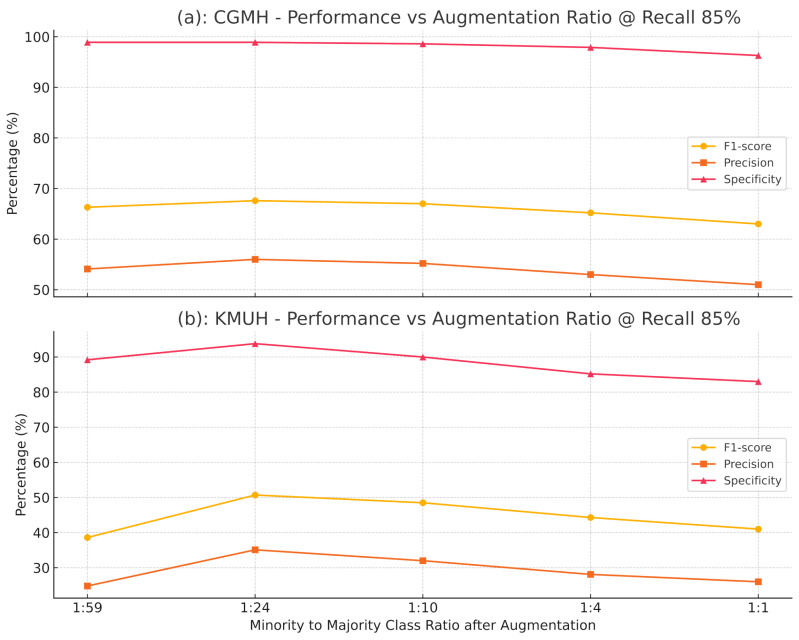
Performance comparison across different augmentation ratios on CGMH and KMUH datasets (recall = 85%).

To assess the influence of augmentation ratios on model performance, we conducted a controlled analysis using a fixed configuration of Euclidean-based CBU and CTGAN. The positive-to-negative sample ratio was systematically varied across five levels: 1:59, 1:24, 1:10, 1:4, and 1:1. As shown in Figure 5, increasing the augmentation ratio to 1:24 led to notable improvements in F1-score, precision, and specificity across both the CGMH and KMUH datasets. However, further increasing the ratio to 1:4 or 1:1 resulted in declining performance, accompanied by a noticeable drop in recall. This suggests that excessive synthetic data may cause overfitting or introduce noise into the decision boundaries. Overall, the 1:24 ratio provided the most favorable balance between predictive accuracy and model robustness, and was thus selected as the default augmentation setting in our final model configuration.

### 3.4. Quality of Synthetic Data

To evaluate the fidelity of synthetic data relative to the original dataset, we employed two complementary metrics: Maximum Mean Discrepancy (MMD) and Pairwise Correlation Difference (PCD). These metrics capture global distribution similarity and inter-variable structural integrity, respectively. The results for each augmentation method are presented in Table 9.

**Figure 6:**
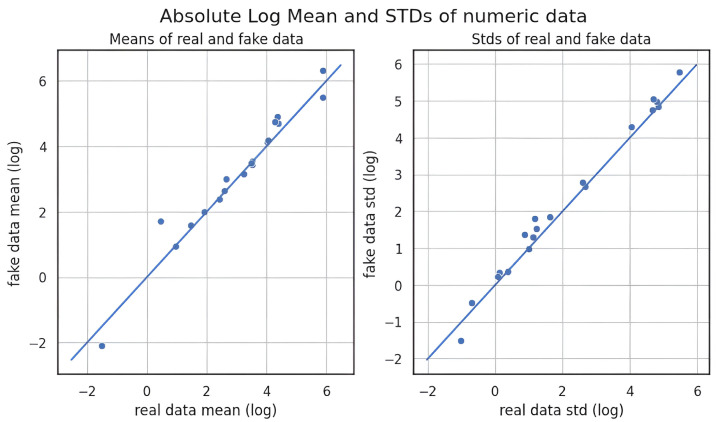
Comparison of feature-wise distributions between real and synthetic data generated by KDE.

**Figure 7:**
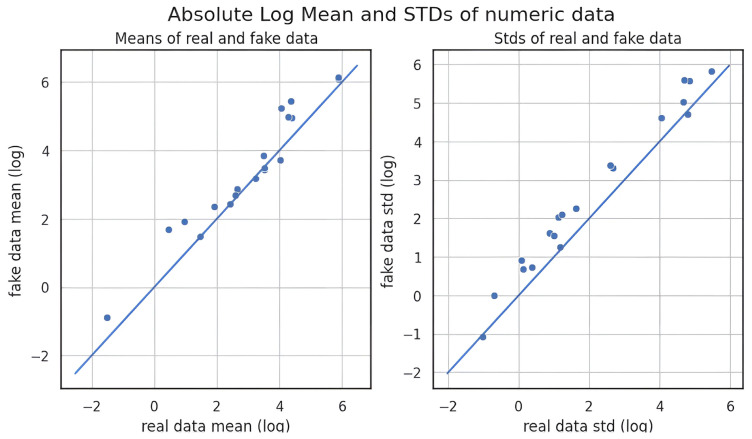
Comparison of feature-wise distributions between real and synthetic data generated by CTGAN.

**Figure 8:**
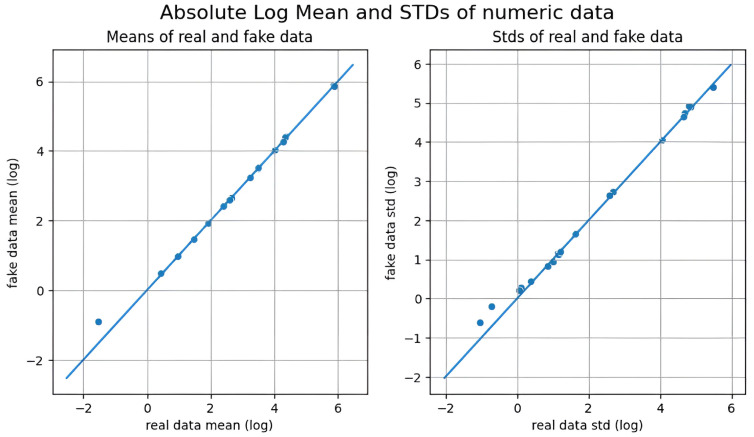
Comparison of feature-wise distributions between real and synthetic data generated by GANBLR.

All three methods achieved nearly identical MMD scores, indicating comparable alignment with the original data distribution at the global level. However, KDE consistently outperformed CTGAN and GANBLR in both metrics, suggesting superior preservation of both marginal distributions and variable relationships. In contrast, the slightly elevated PCD values of CTGAN and GANBLR imply increased structural variability in their augmentation data outputs. A qualitative overview of these results is provided in Table 10, where star ratings were assigned by ranking each augmentation method per metric and mapping the results to a five-level ordinal scale (from ★ to ★★★★★). This simplified representation enables rapid visual comparison. Feature-wise visual analyses in Figure 6, Figure 7 and Figure 8 further substantiate these findings: KDE-generated samples exhibited the closest match to the original feature distributions, while CTGAN and GANBLR introduced moderate deviations in select variables.

### 3.5. Feature Informativeness

We quantified how our data-processing pipeline affected the information content of key clinical features by measuring changes in entropy and information gain (IG). Table 11 reports the entropy and IG values for five important features, both before and after applying CBU combined with GANBLR.

After processing, all five features—ALT, AST, CRP, Band, and UWBC—exhibited a substantial increase in entropy, reflecting greater diversity and complexity in the sample distributions. IG values also improved consistently, with ALT and UWBC showing the most significant gains, reaching 0.09320 and 0.09408, respectively. Importantly, these same features were later identified by SHapley Additive exPlanations (SHAP) [30] analysis (Section 4.4) as among the most influential predictors in the final model. The convergence between statistical enrichment and model-based attribution reinforces the effectiveness and robustness of our preprocessing strategy.

## 4. Discussion

In this study, we propose an artificial intelligence framework comprised of three sequential stages: conditional CBU, generative data augmentation, and stacking ensemble learning. This approach addresses the persistent problem of class imbalance in the clinical prediction of KD and aims to deliver high recall and robust generalization across heterogeneous datasets from different medical institutions. In the following sections, we review the model’s performance, evaluate synthetic data quality, assess the influence of various augmentation ratios, and discuss the study’s limitations.

### 4.1. Model Performance and Generalization Across Institutions

As previously mentioned, our three-stage framework delivered robust findings on both CGMH (internal test set) and KMUH (external validation set). With GANBLR augmentation, the model achieved an F1-score of 61.4% on the CGMH test set and improved from 7.54% to 41.3% on the KMUH validation set (Table 12). At a recall threshold of 85%, performance increased to 67.6% for CGMH and to 50.7% for KMUH (Table 4), outperforming both augmentation-only and original-data configurations.

**Table 12:** Comparison of F1-scores across different modeling strategies (Recall ≥ 90%).

Strategy	CGMH (Internal Test Set)	KMUH (External Validation Set)
Baseline Model (XGBoost)	50.3	7.54
Data Augmentation Only (GANBLR)	64.1	34.3
Clustering-Based Augmentation (GANBLR)	61.4	41.3

To further investigate model performance under varying sensitivity demands, we analyzed how precision changed as recall thresholds increased from 80% to 95% (Table 13). As expected in imbalanced classification settings, precision declined with higher recall—dropping from 63.6% to 37.3% on CGMH and from 33.0% to 13.3% on KMUH—reflecting the inherent trade-off between reducing false negatives and managing false positives. Notably, specificity remained consistently high across thresholds, ranging from 97.5% to 99.3% on CGMH and from 74.7% to 92.7% on KMUH.

**Figure 9:**
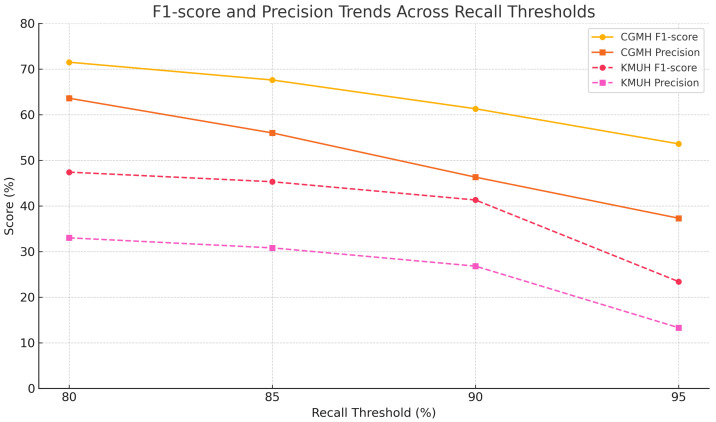
F1-score and precision across varying recall thresholds on internal (CGMH) and external (KMUH) datasets, illustrating performance trade-offs and generalization trends.

To complement the tabular findings, Figure 9 visualizes the trends of F1-score and precision across increasing recall levels. Both the CGMH and KMUH datasets exhibit a smooth, parallel decline in performance, underscoring the model’s generalization capacity and the absence of overfitting. Although the drop in precision is more pronounced on the KMUH dataset—a pattern commonly observed under strong sensitivity constraints—the absence of abrupt drops or divergence between curves confirms stable behavior across institutions, even with a limited number of KD cases in the external validation cohort.

Under a fixed recall of 90%, the GANBLR-based augmentation strategy yielded the highest F1-scores on both the CGMH (61.4%) and KMUH (41.3%) datasets (Table 14), outperforming CTGAN and KDE. While variability on the KMUH dataset is partly attributable to its small sample size, the results emphasize the robustness and cross-site adaptability of the proposed model.

The stacking ensemble architecture, integrating XGBoost, LightGBM, and AdaBoost as base learners and logistic regression as a meta-classifier, further achieved an AUC of 83.7% on CGMH (Table 15) [24,25,26]. These findings affirm the synergistic advantage of integrating sample filtering, synthetic data augmentation, and ensemble learning for scalable KD screening. To elucidate the observed performance differentials across Table 4, Table 5, Table 6 and Table 7 and Table 11, Table 12, Table 13, Table 14, Table 15, Table 16, Table 17 and Table 18, we interpret the results in light of our experiments and the prior literature.

In the CBU phase, Euclidean distance consistently yielded the most stable results across both CGMH and KMUH (Table 7), likely due to its superior ability to preserve global geometric structures in high-dimensional clinical data. Conversely, Manhattan distance—due to its axis-aligned nature—may underperform in capturing subtle distributional nuances [18,19].

**Table 15:** Performance of the stacking ensemble model by base learner composition and final integration.

Model Combination	Base Learners	F1-Score (%)	AUC (%)
XGBoost Only	XGBoost	50.3	76.5
Ensemble (w/o Augment)	XGBoost+LightGBM+AdaBoost	54.8	79.1
Full Model (w/CBU+GANBLR)	XGBoost+LightGBM+AdaBoost	61.4	83.7

During the augmentation phase, method-specific variations emerged. At a recall threshold of 85%, CTGAN slightly outperformed GANBLR on KMUH (F1: 50.7% vs. 45.3%), though both performed equally well on CGMH (F1 = 67.6%). However, under stricter constraints (Recall ≥90%), GANBLR proved more effective, yielding higher F1-scores on both datasets (Table 14). Prior work supports GANBLR’s capacity to enhance generalization by improving information gain and refining decision boundaries (Table 11).

While CTGAN offered more balanced performance on KMUH under relaxed constraints, our objective was to prioritize recall in order to reduce missed diagnoses. Accordingly, our final configuration featured Euclidean-based CBU, a 1:24 augmentation ratio, and a stacking ensemble combining XGBoost, AdaBoost, and LightGBM, with logistic regression as the integrator. Though both CTGAN and GANBLR reached F1 = 67.6% on CGMH at 85% recall, only CTGAN achieved F1 = 50.7%, PPV = 35.1%, and specificity = 93.8% on KMUH. Under more stringent recall settings, however, GANBLR consistently outperformed CTGAN. Thus, GANBLR was selected as the augmentation method of choice, offering superior reliability and cross-institutional generalization for early KD screening.

### 4.2. Balancing Data Augmentation Quality and Predictive Performance

Effective handling of class imbalance is essential for robust clinical prediction and was implemented via conditional CBU. On the KMUH validation set at a recall threshold of 90%, CBU with Euclidean distance achieved an F1-score of 35.7% (Table 16), compared with 20.3% for conventional random undersampling, demonstrating enhanced cross-institution generalization.

**Table 16:** Comparison of undersampling methods on the KMUH validation set (recall ≥ 90%).

Undersampling Method	KMUH F1-Score (%)
Random Undersampling	20.3
Clustering-Based (Euclidean)	35.7

To evaluate generative data augmentation, we assessed statistical fidelity using MMD and PCD [21,22] and compared downstream performance in Table 17. As illustrated in Figure 6, Figure 7 and Figure 8, all three methods produced data distributions that closely mirrored those of the original samples, reflected by uniformly low MMD and PCD scores. Among them, KDE exhibited the lowest MMD and PCD scores but yielded only modest gains in F1-score as previously shown in Section 3.2 and Section 3.3 and summarized in Table 17. In contrast, CTGAN and GANBLR, despite slightly higher divergence, delivered superior improvements in entropy, information gain, and classification performance on KMUH. For example, as shown in Table 11, synthetic data generated by GANBLR increased the information gain of ALT from 0.025 to 0.093, AST from 0.016 to 0.084, CRP from 0.016 to 0.061, and UWBC from 0.026 to 0.094. Furthermore, GANBLR and CTGAN improved F1-scores by approximately 9.6% and 6.3%, respectively, over KDE on the KMUH set. These findings indicate that statistical similarity alone does not predict predictive utility and underscore the importance of balancing distributional fidelity with learning efficacy. In summary, statistical fidelity alone does not guarantee predictive utility, and augmentation quality should be judged by both distributional alignment and its impact on model performance.

**Table 17:** Statistical and predictive evaluation metrics for augmentation data quality.

Augmentation Method	MMD ↓	PCD ↓	Entropy ↑	Info Gain ↑	F1-Score Improvement (%)
KDE	0.10	0.07	2.51	0.12	2.1
CTGAN	0.18	0.10	2.78	0.21	6.3
GANBLR	0.15	0.06	2.91	0.24	9.6

### 4.3. Impact of Augmentation Ratios on Model Performance

**Figure 10:**
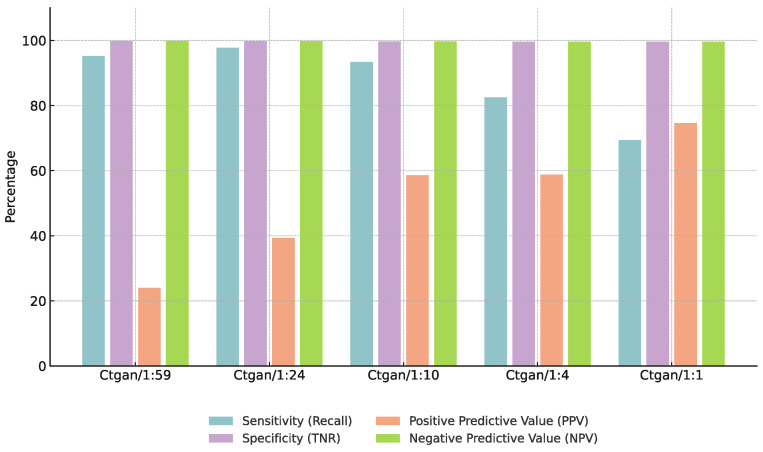
Performance trends at different augmentation ratios (CGMH test set).

**Figure 11:**
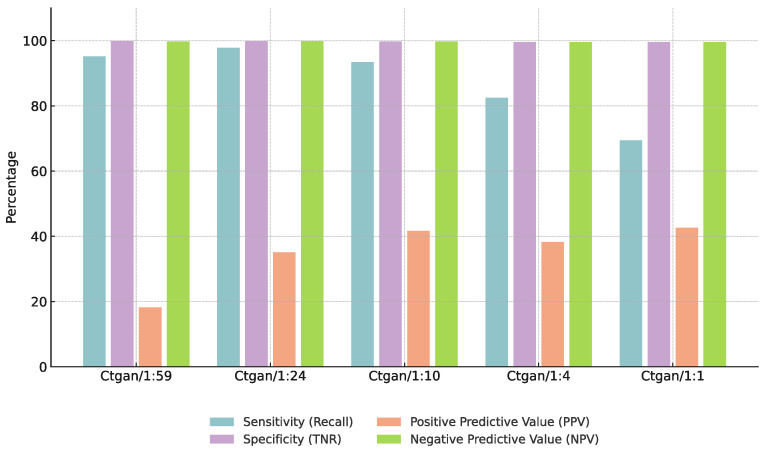
Performance trends at different augmentation ratios (KMUH validation set).

We evaluated five positive-to-negative sample ratios (1:59, 1:24, 1:10, 1:4, and 1:1) using the fixed Euclidean-CBU + CTGAN configuration (Section 3.3). As shown in Table 18 and illustrated in Figure 10 and Figure 11, the 1:24 ratio achieved the highest F1-score, precision, and specificity on both the CGMH and KMUH datasets. When ratios exceeded 1:24, both F1 and recall declined—despite minor precision gains—suggesting overfitting and less distinct decision boundaries (as demonstrated by a 5.2% decrease in F1-score at a 1:4 ratio and an 8.7% decline in recall at a 1:1 ratio on the KMUH dataset).

**Table 18:** Impact of augmentation ratios on model performance.

Augmentation Ratio (Pos:Neg)	F1-Score (%)	Specificity (%)
1:59	50.3	95.0
1:24	61.4	97.2
1:10	58.5	96.8
1:4	55.0	95.5
1:1	48.0	94.0

### 4.4. Study Limitations and Future Directions

As demonstrated in Section 4.1 and Section 4.2, the proposed multi-stage framework consistently achieved strong predictive performance and reliable cross-institution generalization under high-recall conditions. The use of an entirely independent dataset from a separate medical center (KMUH), collected during a non-overlapping time frame, represents a key strength of this study. This form of external validation offers a rigorous test of real-world generalizability, beyond what is attainable through internal cross-validation or random train–test splits. Despite institutional differences and the relatively limited sample size of the KMUH dataset, the framework consistently delivered robust performance, underscoring its potential utility across diverse clinical settings.

Importantly, the confusion matrices presented in Section 3.1 illustrate a consistent decline in precision and specificity when moving from the internal CGMH test set to the external KMUH validation set. This pattern aligns with expected behavior under increasing data heterogeneity and supports the model’s generalization capacity. No evidence of overfitting or data leakage was observed throughout the evaluation process. While confusion matrices for internal and external test sets were reported, we intentionally omitted results from the training set to avoid inflated performance estimates under high-recall constraints. This design choice ensures that the observed performance drop reflects true generalization rather than artifacts of model fitting. Future work may consider including training-level assessments to enable a more complete three-tier generalization analysis.

**Figure 12:**
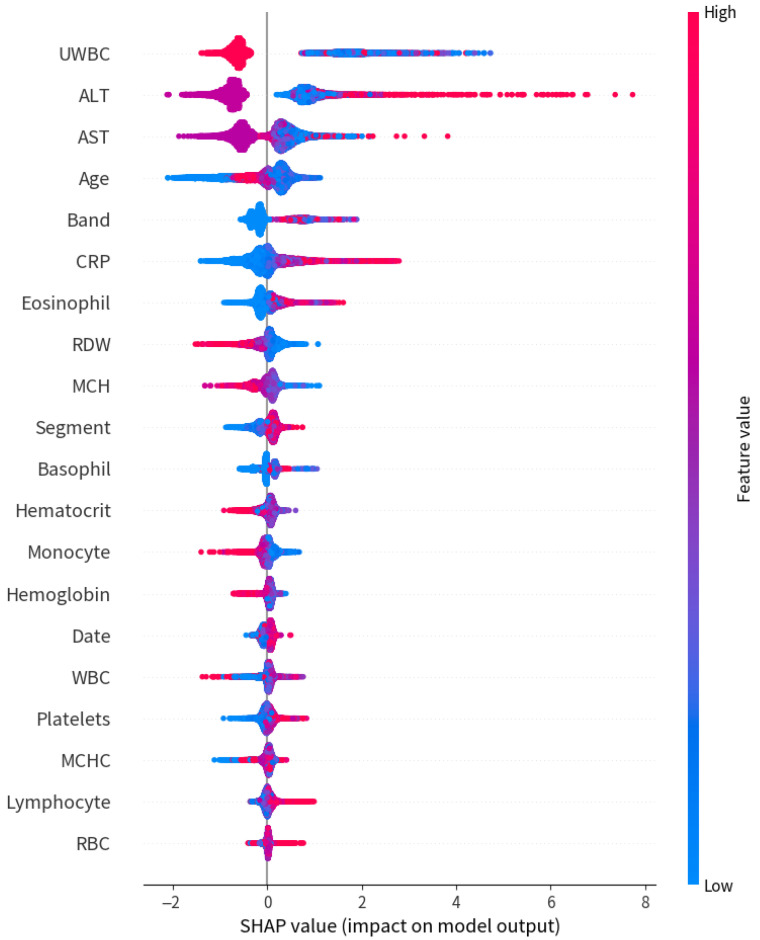
SHAP summary plot of feature importance from the final LightGBM model. The top-ranked predictors included UWBC, ALT, AST, age, and band neutrophils. Each dot represents a patient, with color indicating the feature value (red = high, blue = low), and x-axis indicating its impact on model output.

To enhance interpretability, we performed a post hoc SHAP analysis (Figure 12), which identified UWBC, ALT, AST, patient age, and band neutrophils as the most influential predictors in the final model. These features are strongly aligned with established biomarkers of systemic inflammation and the known pathophysiology of pediatric KD. Notably, their prominence in the SHAP summary plot closely mirrored the top information gain scores observed after data augmentation (Section 3.5), suggesting that the model not only captured statistical patterns but also prioritized clinically meaningful signals. This convergence between model behavior and domain knowledge reinforces both the validity of the preprocessing pipeline and the credibility of the model’s decision boundaries.

Nevertheless, several limitations warrant discussion:

First, although our data were split into training and test sets with five-fold cross-validation, the absence of a dedicated internal validation set may introduce subtle bias during hyperparameter tuning. In datasets of this scale (n≈74,641), a three-way split—comprising separate training, validation, and test subsets—is generally recommended to ensure full independence between model selection and final evaluation. While we mitigated this limitation through validation on a fully independent external dataset (KMUH), future studies should consider employing a dedicated validation subset to further enhance evaluation rigor.

Second, while we assessed the quality of synthetic data in Section 4.2 using statistical (MMD, PCD) and information-theoretic (entropy, information gain) metrics, there remains no universally accepted benchmark for evaluating synthetic clinical data. Although we selected a fixed augmentation ratio of 1:24 based on empirical optimization, its capacity to fully capture clinical heterogeneity—particularly across different institutions—remains uncertain. Future work should explore adaptive augmentation strategies, such as reinforcement learning, and incorporate domain expert validation to dynamically tune augmentation parameters, especially ratio selection.

Third, the external validation cohort from KMUH, while valuable, represents only a single institution and a limited patient population. Broader validation across multiple centers, geographic regions, and healthcare systems is essential to confirm the framework’s robustness and support real-world deployment [20]. This aligns with growing consensus in the field advocating for collaborative benchmarking and multi-institutional model validation to mitigate site-specific biases.

Fourth, the generative models used for data augmentation were unconditional and did not allow targeted synthesis of clinically relevant subgroups. To better support real-world applications, future work should consider conditional generative models or diffusion-based techniques, which may allow for the controlled generation of minority phenotypes or rare diagnostic presentations—thereby enhancing rare-case detection and model fairness.

Fifth, although SHAP was employed for retrospective interpretation during model development, our current system lacks an integrated, real-time explainability module that can support clinical use. To enhance practical usability—especially in rare disease contexts—future iterations should incorporate interactive explainable AI (XAI) components, such as embedded SHAP visualizations, LIME [31], counterfactual explanations [32], or rule-based reasoning models, to facilitate both global model understanding and individualized decision transparency [13,14]. In this context, “real-time, embedded interpretability modules” refer to interfaces that automatically present individualized model explanations at the point of prediction, seamlessly integrated into clinical decision workflows.

Finally, although our model leverages widely available blood and urine test parameters—providing affordability and high clinical accessibility—its integration into routine clinical workflows has yet to be empirically validated. Real-world deployment will require prospective clinical trials, implementation feasibility studies, and user-centered design approaches to ensure that algorithmic innovations translate into actionable clinical tools. Moving forward, it is essential to prioritize interpretability, usability, and seamless integration into clinical systems to bridge the gap between technical development and frontline medical practice.

Overall, the framework developed in this study leverages inexpensive, routinely available blood and urine test indicators and demonstrates robust cross-institutional performance under high-recall settings—achieving F1-score improvements of approximately 22% on CGMH and 71% on KMUH compared to baseline models. It thus lays a practical foundation for deploying AI-based screening in primary care.

### 4.5. Ethical Considerations

The deployment of AI in pediatric medicine—exemplified by the Kawasaki disease prediction framework presented here—must be evaluated not only on technical merit, but also through the lens of ethical responsibility. While this study received ethical approval (IRB-2025-001) and utilized fully anonymized data, broader ethical dimensions surrounding AI deployment in pediatric settings merit explicit attention. Our multi-stage architecture addresses critical challenges like class imbalance and cross-institutional generalization, the inherent sensitivity of pediatric data and the clinical stakes involved necessitate unwavering adherence to ethical standards.

Foremost among these is data privacy. Our strategy leverages synthetic data augmentation to reduce dependence on real patient data, thus preserving confidentiality while supporting effective model training. Yet as AI becomes increasingly integrated into clinical care, there is a growing need for robust ethical governance. Giuffrè and Shung [33] have drawn attention to key tensions in synthetic data research—particularly surrounding data ownership, fairness, and algorithmic transparency. In response, Mehta et al. [34] introduced the PEARL-AI framework, a pediatric-centered ethical guideline that emphasizes transparency, accountability, fairness, and clinical safety.

To align with such principles, future iterations of our model should prioritize interpretability to strengthen clinical trust and enable informed decisions. As we noted in the Limitations section, any clinical implementation should be preceded by prospective trials that undergo thorough ethical review, ensuring fairness and addressing potential biases across diverse patient groups. By integrating ethical reflection throughout the AI development and deployment cycle, we can maximize the clinical utility of models like ours—while upholding the highest standards of safety, equity, and trust.

## 5. Conclusions

This study introduces a robust, multi-stage artificial intelligence framework designed to address the extreme class imbalance prevalent in Kawasaki disease (KD) prediction. By integrating conditional clustering-based undersampling, synthetic data augmentation, and stacked ensemble learning, this framework not only significantly enhances predictive performance but also demonstrates superior generalization capabilities in multi-institutional validation.

The practical utility of this framework lies in its reliance on routine laboratory tests, specifically standard blood and urine indices, ensuring its accessibility and ease of integration into daily clinical workflows, particularly in primary care and resource-limited settings. Furthermore, the employed data augmentation strategy achieves an optimal balance between enhancing minority class representation and preserving decision boundary integrity, effectively improving the model’s sensitivity towards rare KD cases without compromising overall predictive accuracy. Beyond numerical performance gains, this study also highlights the importance of combining synthetic data generation with targeted undersampling methods to effectively tackle the class imbalance challenge inherent in clinical datasets.

Despite the strengths demonstrated in this study, several limitations should be acknowledged, including the reliance on unconditional generative models and the limited sample size from participating institutions. While the model exhibited initial generalizability across two independent medical centers, large-scale prospective studies are essential to validate its broader applicability across diverse clinical settings. Building upon these findings, future research should focus on the following directions:

- Exploring conditional or diffusion-based augmentation for targeted subgroup synthesis;
- Integrating multimodal clinical data (imaging, genomics);
- Advancing the integration of XAI capabilities to support clinician trust and transparent decision-making;
- Conducting prospective, multi-center trials and workflow integration studies.

Overall, the findings of this study underscore the broad applicability potential of such AI-driven approaches in effectively reducing the rates of missed and delayed KD diagnoses, ultimately leading to improved patient outcomes through early intervention and management.

## Figures and Tables

**Table 1:** Feature distribution of the dataset.

Category	Number of Features	Example Features
Demographics	3	Sex, Age, Month of Visit
Blood Tests	17	WBC, Platelet, CRP, AST, ALT
Urine Tests	2	UWBC, Pyuria

**Table 2:** Summary of training, test, and validation sets.

Dataset Type	Positive Samples	Negative Samples	Pos/Neg Ratio
Training Set	913	58,799	1:64
Test Set	229	14,700	1:64
Validation Set	62	1520	1:24

**Table 5:** Confusion matrix results for GANBLR + Stacking on CGMH and KMUH test sets.

Dataset	TP	FP	FN	TN
CGMH	195	153	34	14,547
KMUH	53	119	9	1401

**Table 6:** Comparison of CBU and RUS model performance (recall = 95%).

Undersampling Method	F1-Score (%)	Precision (%)	Specificity (%)
CBU + CTGAN + Stacking (CGMH)	50.6	34.4	97.2
RUS + CTGAN + Stacking (CGMH)	48.8	32.8	97.0
CBU + CTGAN + Stacking (KMUH)	26.8	15.6	79.0
RUS + CTGAN + Stacking (KMUH)	14.3	7.7	53.5

**Table 7:** Comparison of clustering distance metrics (recall = 85%).

Distance Metric	F1-Score (CGMH, %)	F1-Score (KMUH, %)
Euclidean	67.6	50.7
Manhattan	64.1	34.4
Cosine	64.9	35.7

**Table 8:** Comparison of data augmentation methods (1:24 ratio, recall = 85%).

Augmentation Method	F1-Score (CGMH, %)	F1-Score (KMUH, %)
CTGAN	67.6	50.7
KDE	65.0	46.1
GANBLR	67.6	45.3

**Table 9:** Evaluation of synthetic data quality across augmentation methods using MMD and PCD metrics.

Augmentation Method	MMD	PCD
CTGAN	0.0018952004438895825	0.11355271761877538
GANBLR	0.0018952004438895825	0.10377477877428594
KDE	0.0018948196393339823	0.03397027996432563

**Table 10:** Visual summary of similarity between real and synthetic data, based on comparisons of feature-wise means and standard deviations. Star ratings (★) provide an intuitive representation of relative performance across evaluation metrics.

Augmentation Method	Similarity in Feature Means	Similarity in Standard Deviations
**KDE**	★★★★✩ (Highest)	★★★★✩ (Most Stable)
**CTGAN**	★★★★✩ (Moderate Shift)	★★✩✩✩ (Higher Variability)
**GANBLR**	★★★✩✩ (Moderate Shift)	★★✩✩✩ (Higher Variability)

**Table 11:** Comparison of entropy and information gain for key clinical features before and after data processing.

Feature	Entropy (Original)	Entropy (CBU + GANBLR)	IG (Original)	IG (CBU + GANBLR)
ALT	4.72	6.43	0.02472	0.09320
AST	4.51	6.07	0.01579	0.08412
CRP	5.06	5.52	0.01594	0.06060
Band	1.53	2.99	0.00211	0.07852
UWBC	4.01	5.91	0.02645	0.09408

**Table 13:** Model performance under varying recall thresholds at CGMH and KMUH.

Recall Threshold	Dataset	Actual Recall (%)	Precision (%)	Specificity (%)	F1-Score (%)	F2-Score (%)
80%	CGMH	81.7%	63.6%	99.3%	71.5%	77.3%
85%	CGMH	85.2%	56.0%	99.0%	67.6%	77.2%
90%	CGMH	90.8%	46.3%	98.4%	61.3%	76.2%
95%	CGMH	95.2%	37.3%	97.5%	53.6%	72.6%
80%	KMUH	83.9%	33.0%	92.7%	47.4%	64.1%
85%	KMUH	85.5%	30.8%	92.2%	45.3%	63.1%
90%	KMUH	90.3%	26.8%	90.0%	41.3%	56.0%
95%	KMUH	95.2%	13.3%	74.7%	23.4%	42.7%

**Table 14:** Comparison of F1-scores for different data augmentation methods (recall ≥ 90%).

Augmentation Method	CGMH F1-Score (%)	KMUH F1-Score (%)
CTGAN	58.8	35.7
KDE	58.2	35.3
GANBLR	61.4	41.3

## Data Availability

The real medical datasets are not available due to privacy restriction.
